# Mental Health of Holocaust Survivors and Other Older Adults During the COVID-19 Pandemic in Israel

**DOI:** 10.1093/geroni/igab046.773

**Published:** 2021-12-17

**Authors:** Ella Cohn-Schwartz, Yaacov Bachner, Sara Carmel

**Affiliations:** Ben-Gurion University, Beer-Sheva, HaDarom, Israel

## Abstract

Holocaust survivors could be especially vulnerable to the negative effects of the COVID-19 pandemic due to their early life traumas. Thus, the current study examines the effects of the pandemic on the mental health of Holocaust survivors in Israel, compared to adults who did not experience the Holocaust. We collected quantitative data from 305 adults aged 75+ (38% Holocaust survivors) in Israel during the COVID-19 pandemic. The results indicate that Holocaust survivors were worried to a greater extent from COVID-19 and reported greater depression which became worse during the pandemic. On the other hand, despite these differences, the two groups were similar in their will to live. In conclusion, Holocaust survivors seem to be more vulnerable to the COVID-19 pandemic, strengthening the vulnerability hypothesis, while also showing resilience in their will to live. Policy makers and practitioners should pay special attention to this particularly vulnerable population during these difficult times.

